# Single stage repair for aortic root aneurysm in a patient with coexisting coarctation incorporating the Cabrol technique: a case report

**DOI:** 10.1186/s13019-018-0761-2

**Published:** 2018-06-22

**Authors:** Yassir Iqbal, Omar A. Jarral, Pantelis Tsipas, Ilias Samiotis, Theodoros Kratimenos, John Kokotsakis, Thanos Athanasiou

**Affiliations:** 1 Birmingham Children’s Hospital, Birmingham, UK; 2 Imperial College, London, UK; 3 Evangelismos Hospital, Athens, Greece; 4 Department of Surgery and Cancer, Imperial College London, London, W2 1NY UK

**Keywords:** Aortic Coarctation, Aortic root aneurysm, Extra-anatomical bypassCabrol

## Abstract

**Background:**

A 44 year old man who presented with a history of chest pain and dyspnoea was found to have an aneurysm of the aortic root, aortic valve insufficiency, and coarctation of the aorta.

**Case presentation:**

The patient underwent a single stage procedure to treat the aortic root, valve and coarctation with a composite valved conduit and extra-anatomic bypass of the coarctation. The modified Cabrol technique was necessary to attach the coronary buttons due to grossly abnormal anatomy. The patient made a remarkable recovery and was discharged on the 8th post-operative day.

**Conclusion:**

This case report highlights the feasibility and efficacy of performing a single stage procedure on complex coarctation with associated cardiac defects. To the best of our knowledge, this is the first report of the modified Cabrol technique being used in this particular setting.

## Background

Aortic coarctation is a relatively common congenital abnormality. The prevalence accounts for 6–8% of all live births with congenital heart defects. It occurs at the isthmus of the aorta (distal aortic arch between the left subclavian and the site of the ligamentum arteriosus) where there is a discrete narrowing [1].

Patients with associated arch hypoplasia are at increased risk of developing hypertension. They have an increased incidence of acute cardiac events and delayed aneurysm and dissection formation even following correction [2]. There has been much debate as to the optimum surgical strategy to treat this condition. Meticulous preoperative planning is required with thorough multidisciplinary discussion.

We report a case of a successful single stage repair of a complex aortic coarctation with concomitant root aneurysm, and aortic valve insufficiency.

## Case presentation

**Fig. 1:**
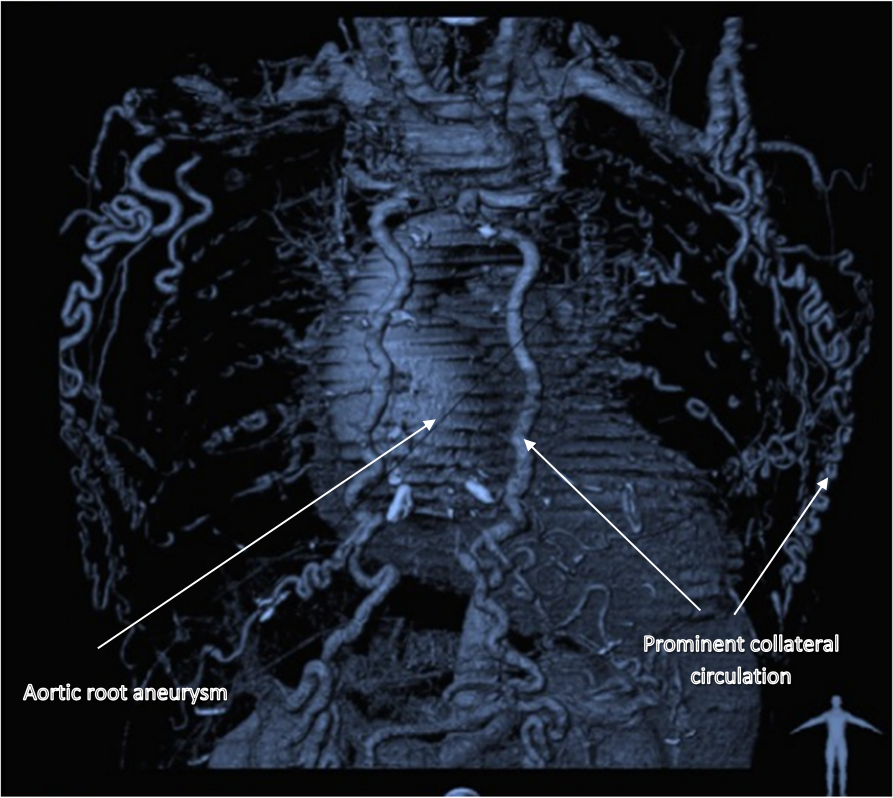
Computer generated 3D reconstruction of aorta. Demonstrating extensive collateral network

A 44-year old male was referred following investigation for chest pain and dyspnoea. He had no pre-existing co-morbidities. Physical examination revealed feeble femoral pulses and he was found to be hypertensive with marked differences between the upper and lower limbs (systolic blood pressure upper limb 190mmmHg, lower limb 75 mmHg, with an ankle brachial index (ABI) of 0.39). Electrocardiogram revealed evidence of severe left ventricular hypertrophy. This was confirmed with echocardiography which also demonstrated a tricuspid aortic valve with significant aortic regurgitation in the presence of an aortic root aneurysm of approximately 9 cm. Left ventricular function was preserved. Computerised tomography angiography (CTA) was performed to evaluate the aortic pathology in further detail (Fig. 1). The scan noted an aortic root aneurysm (8.8 cm), in addition to the presence of severe aortic coarctation, with subtotal occlusion and a lumen less than 6 mm in size. The coarctation was just distal to the left subclavian artery, at the aortic isthmus. There was clear evidence of collateral circulation to the descending thoracic aorta via the subclavian and intercostal arteries. Coronary angiography confirmed a right dominant coronary system with no significant coronary disease.

A multidisciplinary team meeting took place and a consensus was agreed to proceed with a two staged hybrid approach, with the first phase involving an endovascular approach to stent the coarctation, followed by a second stage to perform the surgical repair of the aortic root aneurysm. The first stage to stent the coarctation was unsuccessful via the femoral approach, as the guidewire could not cross the coarctation. Assessment through angiography via the left brachial artery showed complete obstruction at the aortic isthmus. The decision was then made to proceed to a single stage surgical approach to treat both lesions.

After induction of anaesthesia, arterial lines were placed in the left radial and left femoral artery. A right infraclavicular incision and a right groin incision was made this was to establish peripheral arterial cannulation access to the right axillary and right femoral artery. An 8 mm dacron graft was anastomosed to each vessel for indirect cannulation. Median sternotomy was performed to access the mediastinum and expose the heart and aorta. Following heparinisation cardiopulmonary bypass (CPB) was established with venous return from bi-caval cannulation. The body temperature was cooled to 25 degrees Celsius. The right superior pulmonary vein was used for venting. Once the cross clamp was applied, complete cardiac arrest was achieved using Custodiol 25 ml/kg crystalloid cardioplegia via a retrograde cannula through the coronary sinus. A further top up of cardioplegia was given once the aorta was opened through direct cannulation of the coronary ostia.

**Fig. 2:**
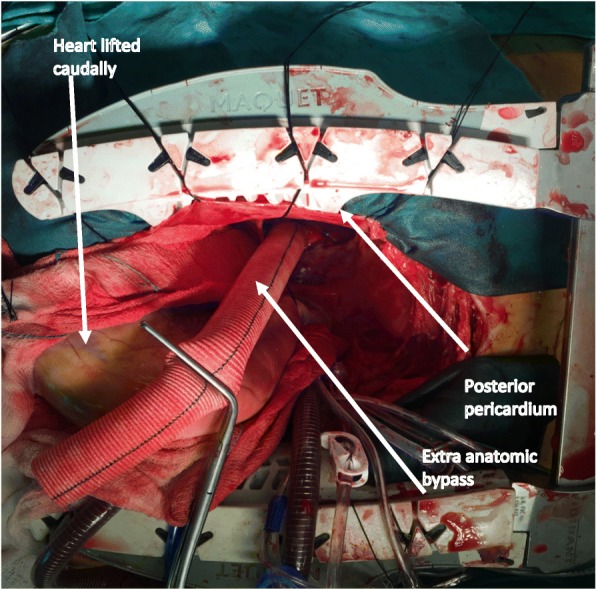
Surgeons view; longitudinal incision in posterior pericardium to anastomose extra-anatomical bypass to DTA

The aortic root, valve and ascending aorta were excised. The coronary ostia were fashioned as buttons from the native aortic root. The coronary ostia were noted to be significantly displaced, with distorted anatomy due to the patient’s disease process. Therefore, 8 mm dacron grafts were attached end-to-end to each ostia, with view to performing the modified Cabrol technique later following replacement of the root. The heart was then retracted in a cephalad position to access the posterior pericardium. A vertical incision was made to expose the descending thoracic aorta (DTA). An end to side anastomosis was formed with a 20 mm dacron graft to the DTA (Fig. 2). This graft was then routed posterior to the inferior vena cava (IVC) and anterior to the right inferior pulmonary vein (RIPV), adjacent to the right atrium (RA). Root replacement was then performed with a 25 mm biological valved-conduit, as this was favoured by the patient over a mechanical prosthesis, despite the risk of a difficult redo procedure in the future. The 8 mm dacron grafts attached to the coronary ostia were anastmosed to the root conduit as neo coronary ostia. The distal part of the valved-conduit was anastomosed to the proximal arch under selective antegrade cerebral perfusion (SACP). Finally, an end to side anastomosis was fashioned between the 20 mm extra-cardiac graft (attached to the descending thoracic aorta) and the ascending portion of the valved-conduit. Valve-sparing root replacement was not considered in this patient due to the grossly abnormal aortic anatomy.

**Fig. 3:**
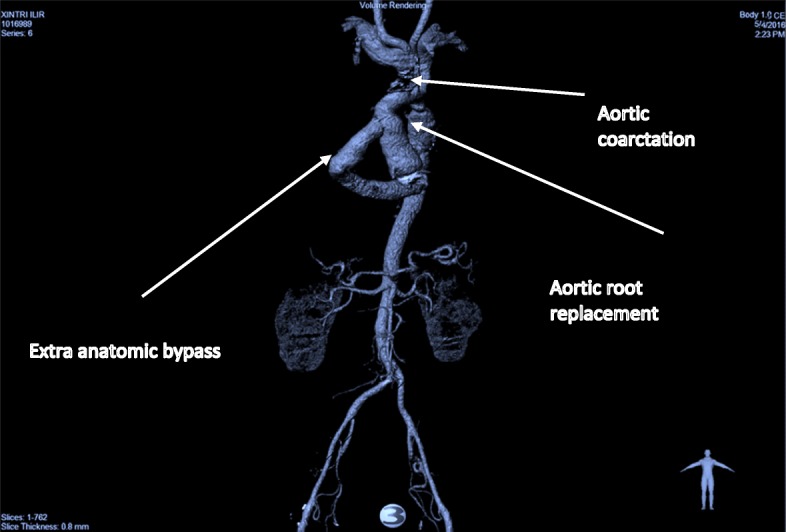
Computer generated 3D reconstruction demonstrating surgical repair

Following rewarming and deairing the patient was successfully weaned off CPB. The bypass time was 160 min, the cross-clamp time was 120 min, and the SACP time was 40 min. Haemostasis was achieved and thereafter a routine closure of all incision sites. The patient remained in ICU for less than 48 h, and made excellent progress on the ward. Minimal anti-hypertensives were required and the patient was discharged on 8th day post operatively neurologically intact and independent. At 3 months follow up the patient underwent a repeat CTA scan which showed complete patency in the extra-anatomical graft and resolution of the collateral arterial network (Fig. 3).

## Discussion

Aortic coarctation is a common congenital cardiac defect, with narrowing of the aorta at the isthmus. The condition is generally identified and treated during infancy and childhood. However presentation of untreated aortic coarctation in adults with associated cardiac defects are scarcely reported [3, 4].

In this complex case we treated three distinct lesions (aortic root aneurysm, aortic valve insufficiency and aortic coarctation) through a single staged approach. An additional challenge encountered was the reimplantation of the coronary buttons. The aortic root aneurysm was of a significant size, hence reimplantation of the coronary ostia directly on to the neo aorta was not possible as the coronary arteries would be overstretched. The use of the modified Cabrol technique was incorporated into this single staged operation to provide safe implantation of the coronary buttons with a tension free anastomosis [5]. We believe this is the first reported case where the modified Cabrol technique has been used in this setting.

There remains a wide range of treatment options for aortic coarctation which can be broadly divided into surgical and non-surgical strategies. Non-surgical interventional techniques are becoming increasingly popular, either with balloon angioplasty and or insertion of a stent. These approaches can be used both in native and recurrent disease. However, there is insufficient long-term data available in comparing these interventions with surgical repair. Although these approaches are often associated with lower morbidity compared to surgery, there is an increased rate of re-stenosis 11% versus 2% [6, 7] hence a higher need for reintervention. Reintervention may also be required due to stent fracture, migration and possible aneurysm formation. Careful patient selection is required as not all cases are amenable to percutaneuous intervention. Our patient underwent an interventional approach initially, however the guidewire was unable to cross the lesion.

Surgical repair can include the option of primary repair of the coarctation, either through a left thoracotomy (2-stage repair when in the presence of other cardiac defects) or median sternotomy. However, this is associated with many challenges especially in the adolescent and adult patient population. Extensive mobilisation of the aorta is required, in addition to control of the extensive collateral blood vessels this can contribute to increased risk of bleeding. Other complications can include possibility of lung parenchymal injury, damage to the recurrent laryngeal and or phrenic nerves, chylothorax and the possibility of spinal cord ischaemia [8]. Due to these many risks primary anatomic repair of the coarctation has been avoided in adult patients.

In attempt to reduce complexity of open surgical repair, variations of extra-anatomic bypass have been developed. The single stage procedure was first described by Vijayanagar and colleagues in 1980 [9]. The procedure incorporated an extra-anatomic bypass from the ascending aorta to the DTA through the posterior pericardium. This operation allowed bypass of the coarctation in addition being able to correct other concomitant cardiac defects, i.e. valve replacement, in a single staged approach [8]. This strategy is also preferred in cases where there is a complex coarctation or re-coarctation where a primary repair would be associated with major complications as described above. In addition to the major benefit of being able to treat multiple cardiovascular disorders at the same time in one sternotomy, there is no need for laparotomy to reach the DTA, and the graft is routed in a short course around the right margin of the heart without the risk of compressing the right atrium or ventricle [8].

The initial procedure by Vijayangar described routing the extra anatomic bypass around the left lateral border of the heart. Powell et al. modified this procedure by positioning the graft along the right sided margin of the heart, the benefit being that the graft remains in a more posterior position hence allowing safer re-entry into the chest if reoperation is required [8]. In addition positioning the graft anterior to the RIPV and behind the IVC prevents compression to the right atrium and ventricle [10]. The possible difficulties in this procedure are that accessing the DTA through the posterior pericardium is difficult and exposure maybe more challenging in patients who are obese or have a barrel shaped thorax. The procedure may not be suited to patients in the adolescent group due to their potential somatic growth that could lead to anastomotic dehiscence. Potential long term complications that have been described with this procedure include narrowing of the graft, neointimal thrombus formation, pseudoaneurysm formation or anastomotic dehiscence in patients with who have had somatic growth since their initial procedure. However, in a paper by Connolly et al. [8] with a mean follow up of 3.7 years there were no complications in patients who underwent single stage surgical treatment.

## Conclusion

When coarctation or re-coarctation is associated with cardiac defects that require repair, a single stage approach using cardiopulmonary bypass and coarctation bypass grafting through the posterior pericardium is a safe surgical alternative, as demonstrated by this case. We understand this is also the first reported case where the modified Cabrol technique has been incorporated with this single staged procedure to treat aortic root aneurysm and aortic coarctation. The surgical management of patients with complex coarctation or recoarctation with or without associated cardiac disorders must be individualised and treatment options should be discussed in an open multidisciplinary platform.

Extra-anatomic coarctation bypass is a safe alternative method when endovascular interventions are not feasible. Particular attention must be paid to ensuring the extra-anatomic bypass conduit is well positioned and of a significant calibre. Kinking of this conduit can lead to turbulent blood flow and abnormal wall shear stress which could impact on long term patency. We felt that routing the extra-anatomic bypass posterior to the IVC achieved the best haemodynamic result.

Our strategy has proved to be particularly useful in an adult patient where simultaneous intra cardiac repair is required.

## Abbreviations

ABI: Ankle brachial pressure index
CPB: Cardiopulmonary bypass
CTA: Computerised tomography angiography
DTA: Descending thoracic aorta
ICU: Intensive care unit
IVC: Inferior Vena Cava
RIPV: Right inferior pulmonary vein
SACP: Selective antegrade cerebral perfusion
